# Development of a qPCR Tool for Detection, Quantification, and Molecular Characterization of Infectious Laryngotracheitis Virus Variants in Chile from 2019 to 2023

**DOI:** 10.3390/ani15111623

**Published:** 2025-05-31

**Authors:** Tomás Gatica, Sebastián Salgado, Humberto Reyes, Carlos Loncoman

**Affiliations:** 1 Laboratorio de Virología Molecular, VirionLab, Instituto de Bioquímica y Microbiología, Facultad de Ciencias, Universidad Austral de Chile, Valdivia 5090000, Región de Los Ríos, Chile; 2 Departamento de Genética Molecular y Microbiología, Facultad de Ciencias Biológicas, Pontificia Universidad Católica de Chile, Santiago 8331150, Region Metropolitana, Chile

**Keywords:** tissue culture origin (TCO) vaccines, infectious laryngotracheitis virus (ILTV), molecular characterization, molecular epidemiology

## Abstract

Infectious laryngotracheitis virus (ILT) is the pathogen responsible for a respiratory ailment that has resulted in significant economic losses in the poultry industry, primarily due to high morbidity and mortality rates, as well as diminished egg production. However, for small producers living near the coast, where backyard flocks are located in rural areas, respiratory illness may pose a public health risk rather than an economic one, which must be considered in the differential diagnosis of ILT. Therefore, in this research, we focused on developing an efficient tool to detect, quantify, and classify infectious laryngotracheitis virus (ILTV) field variants. Our results demonstrates that qPCR detected positive samples collected between 2019 and 2023 in flocks exhibiting clinical signs of respiratory illness in Valdivia, in the Los Ríos Region of Southern Chile. Furthermore, the molecular characterization of positive samples using dideoxynucleotide sequencing revealed that the detected viruses were similar to tissue culture origin (TCO) vaccines, even though the birds had never been vaccinated. To the best of our knowledge, this is the first report characterizing ILTV variants in Chile using a molecular approach. Our findings indicate that the tool is useful for detecting ILTV and can also be used to quantify viral particles using a standard curve, making it a valuable tool for the differential diagnosis of respiratory pathogens in poultry.

## Simple Summary

In this study, we investigated a respiratory disease affecting chickens in southern Chile. Although official records have not reported any cases since 2011, this likely reflects a lack of control and monitoring rather than the actual absence of the disease. Local veterinarians suspected infections in backyard chickens that had never been vaccinated. We examined samples from these birds and found clear evidence of the virus. Our analysis showed that the virus was genetically similar to strains used in vaccines, raising concerns about the unintentional spread of vaccine-related viruses through indirect contact, such as contaminated equipment or exposure to the environment. These findings suggest that the disease may be silently circulating in small, non-commercial flocks that are not included in routine surveillance. Using a laboratory technique to detect and analyze the virus’s genetic material, we identified which strains were present and how they might have spread. Our results highlight a gap between official data and actual field conditions. Strengthening disease monitoring in backyard systems could help uncover hidden outbreaks, guide more effective vaccination strategies, and improve poultry health and biosecurity across the country.

## 1. Introduction

Infectious laryngotracheitis (ILT) affects the health, well-being, and sustainability of poultry worldwide. ILT causes an acute and highly contagious respiratory disease in chickens, characterized by conjunctivitis, dyspnea, gasping, tracheitis, and the expectoration of bloody mucus in severe cases [1]. The disease affects large industries and small backyard flocks by decreasing weight gain and egg production and increasing mortality and susceptibility to other respiratory pathogens [2]. The etiological agent of ILT is *Iltovirus gallidalpha 1* (ILTV), which belongs to the genus *Iltovirus*, subfamily *Alphaherpesvirinae*, family *Orthoherpesviridae*, and order *Herpesvirales* [3]. The virus replicates rapidly in epithelial tissues and establishes lifelong latent infection in the peripheral nervous system of the host, which is a key challenge for disease control [4]. The virions are enveloped with an icosahedral capsid containing a linear double-stranded DNA genome approximately 140–150 kbp in length, with a typical type D genome arrangement consisting of unique long (UL) and unique short (US) regions. The US region is flanked by inverted internal repeat (IR) and terminal repeat (TR) sequences [5]. Within the IR region, three open reading frames (ORFs) have been described, with one encoding the transcriptional regulator infected cell protein 4 (ICP4), which is 4482 bp in length and contains recombination hotspot sites [6]. Epidemiological surveillance, biosecurity, and vaccination are the tools used to control ILT worldwide. There are two types of commercially available vaccines: live attenuated vaccines and recombinant viral vector vaccines. Live attenuated vaccines, also known as first-generation vaccines, have been available since the 1960s. These vaccines are either attenuated by serial passages in embryonated eggs, also known as chicken embryo origin (CEO), or obtained by multiple passages in tissue culture, also known as tissue culture origin (TCO) [7]. Although these vaccines have been proven to produce robust protection, some negative effects have been described, such as their ability to infect non-vaccinated birds, revert to virulence, and produce latently infected carrier birds [8,9], suggesting that vaccines may be associated with, and even contribute to, ILT outbreaks worldwide [10]. Furthermore, virulence reversion and subsequent increases in virulence have been observed after serial in vivo passages and promote evolution through recombination, leading to new epidemiologically relevant viral variants [11]. ILT cases originating from live attenuated vaccines have been reported in Italy [12], Northern Ireland [13], Slovenia [14], Korea [15], China [16], Taiwan [17], India [18], Australia [11,19], Canada [20], Argentina [21], Peru [22], and Brazil [23]. In South America, the virus has been detected since 1974, and a detailed review focusing on South American countries has been published elsewhere [24]. Recently, highly similar (genetic distance of ~2%) *Iltovirus gallidalpha*-like sequences were detected using nested PCR and the sequencing of cloacal swabs in samples obtained from Valdivia, Región de Los Ríos, Chile [25]. The sequence identified by Verdugo et al. (2019) [25] in the *Neotropic cormorant* is closely related to a viral variant that was first detected in China [26] and later in Peru [22], and both were submitted to NCBI under the accession numbers JX458822 and MG775618, respectively. Moreover, in 2022, researchers found that viral variants detected in Brazil are closely related to TCO vaccines [23].

In response to these frequent epizootics related to both CEO- and TCO-derived variants, a new generation of vaccines was developed. Currently, there are two commercially available vaccines: the fowlpox virus (FPV) and the herpesvirus of turkeys (HVT). The FPV vectored vaccine carries the UL32 gene corresponding to glycoprotein B [27]. In contrast, there are two HVT-vectored vaccines: one carrying glycoproteins I and D, and another carrying glycoprotein B [28]. Both vectored vaccines are bivalent vaccines and induce protective immunity against ILT and Marek’s disease (MD). These vaccines are characterized by a lack of bird-to-bird transmission and, most importantly, do not revert to virulence [29]. However, viral vector vaccines have been reported to be less effective than CEO and TCO vaccines in decreasing shedding of the challenge virus [30]. Nevertheless, when applied prior to live attenuated vaccines, viral vector vaccines reduce viral detection, offering a plausible alternative to mitigate potential outbreaks [7].

To address virulence reversion and vaccine-related cases, many studies have applied different experimental approaches for ILTV detection in different epidemiological contexts, including backyard flocks. This type of flock may serve as a relevant reservoir in potential ILTV outbreaks [31]. Nonetheless, as viruses evolve through genetic changes over time, it is important to test for viral variants using locally sequenced viruses. In this study, we aimed to develop a tool to detect and quantify viral particles in samples, based on ICP4 gene amplification through qPCR, followed by sequencing and the molecular characterization of ILTV variants, with the goal of differentiating between TCO, CEO, and wild-type variants infecting backyard flocks in the Los Ríos Region, Valdivia, Chile, exhibiting respiratory clinical signs.

## 2. Material and Methods

### 2.1. Sampling

Fifty samples from the upper respiratory tract were obtained during necropsies performed on backyard flocks with respiratory clinical signs such as coughing, sneezing, and conjunctivitis. Specifically, 5 samples were collected in 2019; 20 in 2020; 10 in 2021; 10 in 2022; and 5 in 2023. Mortality rates in flocks exhibiting respiratory clinical signs ranged from 5% to 30%. None of the flocks had prior vaccination records. Necropsies were carried out in Southern Chile, Comuna de Valdivia, Región de Los Ríos, between July 2019 and December 2023 by qualified independent field veterinary practitioners, following the recommendations of the Institutional Committee for the Care and Use of Animals of the Universidad Austral de Chile. Finally, the samples were submitted to the Laboratorio de Virología Molecular (VirionLab, Valdivia, Chile) for molecular biology and the detection of viruses. The tracheas were placed into transport media consisting of Dulbecco’s Modified Eagle Medium (DMEM) with antibiotic, antimycotic solution 10× (ThermoFisher, Valdivia, Chile). The samples were transported in a cryogenic shipper containing liquid nitrogen and stored at −80 °C. Viral DNA was extracted and purified immediately after sample arrival using a viral DNA/RNA nucleic acid purification kit (Geneaid, Valdivia Chile), following the manufacturer’s protocol.

### 2.2. Virus Detection

To detect positive samples, a fragment of the UL15 gene was amplified using primers previously published by [32] (Table 1). The ICP4 fragments (ICP4-1 and IPC4-2) corresponded to a gene that was previously identified as a recombination hotspot within the ILTV genome [6] and as useful for distinguishing between TCO, CEO, and wild-type viral variants [33]. The designed primers targeting the ICP4 coding sequence were named ICP4-1 and ICP4-2 (Table 1), generating amplicons of 688 bp and 635 bp, respectively. The Brilliant II SYBR Green (Agilent Technologies, Valdivia, Chile) kit was used, following the manufacturer’s instructions. Briefly, a 20 µL mixture containing 0.3 µL of each forward and reverse primer (final concentration of 750 nM), 10 µL of the Brilliant II SYBR Green mix (Agilent Technologies, Valdivia, Chile), 4.4 µL nuclease-free distilled water, and 5 µL of DNA template was incubated using the Stratagene Mx3000 qPCR Thermal Cycler (Stratagene, Valdivia, Chile). First, an initial hold at 50 °C for 2 min, followed by 95 °C for 10 min, was performed. Then, 40 cycles of 95 °C for 15 s plus 60 °C for 1 min were completed [32]. Standard curves were generated using a 10-fold serial dilution of linearized DNA, covering eight concentration points. To display the standard curve, the fluorescence data were extracted from the qPCR software and loaded in RStudio v2024.04.2 using the qPCRtools v1.01 package [34].

### 2.3. Cloning and Virus Quantification by Using qPCR

Each gene fragment was cloned into the pJET1.2 vector using the CloneJET PCR cloning kit (Thermo Fisher, Valdivia, Chile). Fragment ligation involved 1 µL of PCR product (amplified using a proofreading polymerase), following the established protocol. Then, 100 µL of DH5-alpha *E. coli* stock was transformed by adding 1 µL of ligation product diluted in Tris-HCl pH 7.5 and 1mM EDTA. The mixture was then incubated on ice for 30 min, followed by heat shock at 45 °C for 45 s, ending with two minutes of cooling on ice. Finally, 0.9 mL of glucose-rich (S.O.C.) Super Optimal broth medium was added and agitated at 225 rpm for 1 h at 37 °C and then cultured in LB (Lysogeny Broth) agar plates using the streak plate method. Plates were incubated overnight at 37 °C. DNA was extracted from separated colonies, and then qPCR to detect the target sequences was run to determine successful cloning. Extracted DNA was then quantified using a spectrophotometer and DNA copy numbers per microliter were calculated using the length of the pJET1.2 vector (2974 bp) plus the PCR products UL15 (113 bp), ICP4-1 (604 bp) or ICP4-2 (662 bp). Copy numbers and ct values were used to build a linear regression and then Ct values for each sample were used in the equation to determine viral copy numbers in field samples.

### 2.4. Sequence Analyses

The resulting PCR products of the ICP4-1 and ICP4-2 primer sets were submitted to the Austral-*omics* sequencing facility at Universidad Austral de Chile for dideoxynucleotide sequencing. The resulting data were further analyzed and curated using Geneious R11 2023.2.1. Consensus sequences were obtained by mapping the ab1 files to a reference sequence in Geneious using the highest sensitivity settings and up to five iterations. Trimming was performed prior to assembly, followed by manual verification of the electropherogram quality.

### 2.5. Phylogenetic Analyses

Each sequenced positive sample produced an individual consensus sequence, which was subsequently curated using Geneious Prime software v2023.2.1. A Phred quality score cutoff of 20 was applied. The resulting consensus sequences were then aligned with 64 complete ILTV genomes obtained from the NCBI database (Supplementary Table S1). Alignments were carried out with MAFFT v7.490, applying the following parameters: scoring matrix 200PAM, k = 2; gap open penalty: 1.53; offset value: 0.123 [35]. Phylogenetic trees were constructed using the Geneious tree builder. Eight iterations were performed using the Jukes–Cantor genetic distance model, the neighbor-joining method, and bootstrap resampling with 100 replicates. Under the same parameters, consensus trees were constructed. The R package “ggtree” was used to visualize the phylogenetic branch containing the sequences of interest [36].

## 3. Results and Discussion

**Figure 1:**
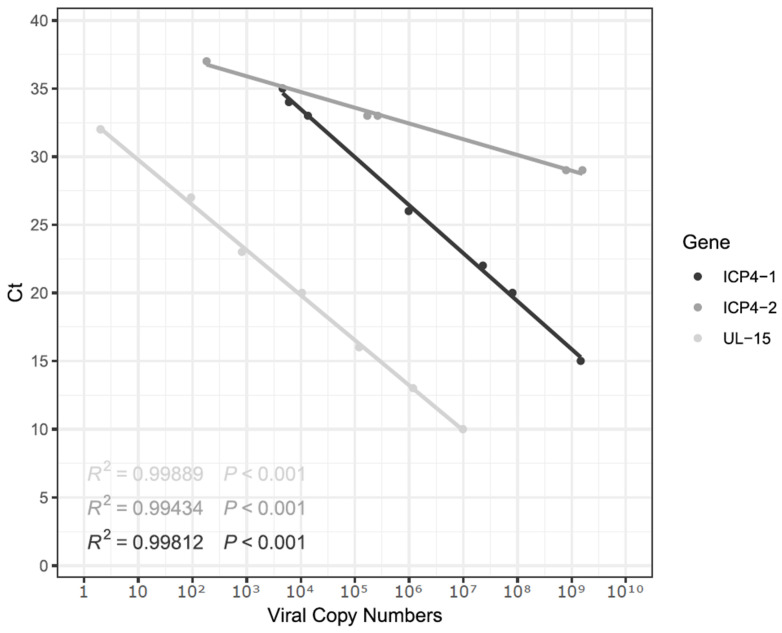
Standard curves were generated using ICP4-1, ICP4-2, and UL15 genes using 10-fold serial dilutions. Efficiencies were 97.62%, 627.89%, and 98.98%, respectively.

**Figure 2:**
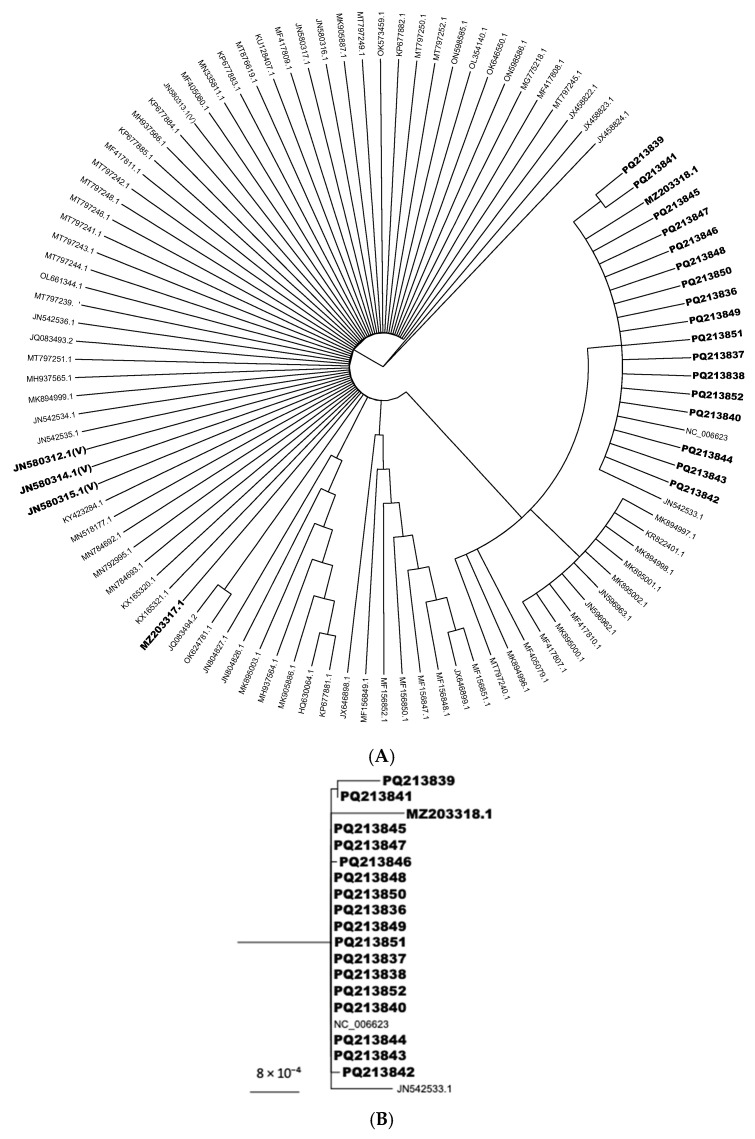
Alignment of the ICP4-1 consensus sequence with 64 ILTV genomes. (**A**) Phylogenetic tree showing the relationship of the ICP4-1 consensus region with 64 other genomes. (**B**) Zoom-in on the clade containing the ICP4-1 consensus sequence, including a scale bar indicating substitution rate. Sequences generated in this study and TCO sequences are shown in bold.

Overall, 45 of the 50 samples were positive for ICP4-1, ICP4-2, and UL15 by PCR, amplifying amplicons of 604 bp, 662 bp, and 113 bp, respectively. Further, 19 sequences were generated from the 45 positive samples. The sequences were assembled and uploaded to GenBank under the following accession numbers (MZ203318.1, MZ203317.1, PQ213836, PQ213837, PQ213838, PQ213839, PQ213840, PQ213841, PQ213842, PQ213843, PQ213844, PQ213845, PQ213846, PQ213847, PQ213848, PQ213849, PQ213850, PQ213851, PQ213852). Ct values for positive samples ranged from 10 to 25, according to the UL15 standard curve (Figure 1). Standard curves using the UL15 and ICP4-1 primers had efficiencies of 97.62% and 98.98%, respectively. In contrast, the ICP4-2 primer had an efficiency of 627.89%. Hence, UL15 and ICP4-1 primer sets were used for detecting viral particles, whereas ICP4-2 was used for sequencing purposes only. All three standard curves had an R-squared value of 0.99. Overall, UL15 primers yielded the best results for viral detection, as previously reported [32]. In this study, samples were submitted to a molecular virology laboratory by field veterinarians to confirm clinical cases of laryngotracheitis in suspected unvaccinated chickens in Valdivia, Los Rios Region, Chile. Using the ICP4-1 primers and a standard curve (Figure 1), we detected and estimated viral genome copy numbers and performed molecular characterization of ILTV variants (Figure 2). ICP4 has been previously detected as a recombination hotspot and has also been used to differentiate viral variants [6,23]. In this study, we used this technique to detect ILTV variants in samples collected from unvaccinated backyard poultry flocks. Our results indicate that the ILTV present in the samples was related to U.S. TCO vaccine strains. Previous studies have attempted similar approaches, targeting other genetic elements, including several glycoproteins encoded by highly conserved regions within the UL and US [37]. Because glycoprotein-related genes are conserved, they may not be ideal targets for the development of molecular epidemiological approaches. However, glycoproteins have proven to be useful in serological screening tests for specific ILTV assessments, which is especially important for differentiating between infected and vaccinated poultry, a relevant aspect to consider when planning control program implementation [7]. The thymidine kinase (TK) gene (i.e., UL23) has also been used to differentiate strains, with diverse results, such as distinguishing field isolates from vaccine strains; however, it does not differentiate between TCO and CEO vaccines [33], nor does it differentiate between TCO and CEO vaccines or between field samples and vaccines [21]. Moreover, TK sequence analysis alone has been used to distinguish wild strains from the SA2 Australian live-attenuated vaccine [10]. In several regions, amplification and the partial sequencing of TK and ICP4 are preferred for ILTV variant differentiation over other genes, such as those encoding glycoproteins [10]. In South America, Chacón and Ferreira (2009) were the first to develop an ICP4 gene amplification and sequencing approach by applying it to samples obtained from severe outbreaks in Brazil and Peru [33]. In their study, the authors found field variants of unknown origin, indicating the possibility of a non-commercial bird trade by small producers in nearby locations. Santander-Parra et al. (2022) recently identified variants between 2015 and 2016 that were phylogenetically related to TCO vaccine strains [23]. These results are similar to those of the present study, suggesting a marked presence of vaccine-derived strains in Brazil and Chile. Furthermore, Craig et al. (2017) found that outbreak-associated sequences in Argentina were CEO-like, even after removing this type of vaccine from the market, due to its severe clinical signs after reversion to virulence [21]. CEO-like variants usually predominate, as they usually display faster replication and spreading than TCO [38]. Similarly to the aforementioned studies, we investigated viral variants in small poultry flocks in southern Chile and found that the ILTV variants described herein were phylogenetically related to TCO vaccines, even though the samples were obtained from unvaccinated backyard birds. Therefore, we hypothesized that at least one indirect transmission event could have occurred through contact with these backyard flocks. This transmission could have been achieved, most likely due to the exposure of backyard birds to contaminated fomites. An alternative hypothesis is that the virus could have been transmitted by other non-replicating hosts, such as the *Neotropic cormorant*. Other authors have found traces of this virus in their virome in the same geographical region [25]. However, herpesviruses are known to be species-specific; therefore, both infection and replication in this host are unlikely.

Molecular analysis was performed by aligning the sequences obtained from positive PCR products that were sequenced by dideoxynucleotide sequencing at Austral-*omics* and curated using Geneious R11 2023.2.1. Minor differences were observed between these nucleotide sequences, indicating a common origin. All consensus sequences generated in this study were aligned with 64 publicly available sequences (Supplementary Table S1) from the United States, China, Australia, Russia, Italy, South Korea, Peru, and Canada. ICP4-1 and ICP4-2 sequences from positive samples displayed ~98% nucleotide identity with sequences including TCO IVAX, TCO high-passage, and TCO low-passage strains, associated with accession numbers JN580312, JN580314, and JN580315, respectively. A nucleotide identity of ~96% was found between sequences from positive samples and the ILTV USDA strain (JN542534) and other sequences derived from the USDA strain, such as USDA09-2019 and USA vModKLO (MN518177 and MN784693). The lack of publicly available data regarding ILT viral genomes in Chile makes it difficult to determine the cause of the predominant presence of TCO-related ILTV. This lack of data also limits our ability to assess whether this scenario is common in other regions of the country, and to draw broader conclusions about the virus’s epidemiology. According to local government veterinary services, ILTV has not been detected since 2011, which contrasts with our findings.

The detection of TCO-like ILTV sequences in unvaccinated backyard chickens raises important questions regarding biosecurity measures, vaccine virus dissemination, and the effectiveness of current surveillance systems in Chile. The presence of vaccine-derived strains in birds with no vaccination history strongly suggests silent environmental circulation, potentially facilitated by the inadequate control of live-attenuated vaccines or poor farm-level containment practices. These findings highlight a gap between official records and actual viral circulation, underscoring the need to update national epidemiological databases and consider routine molecular surveillance, especially in non-commercial settings. The discrepancy between our results and the official reports indicating the absence of ILTV cases since 2011 points to a probable underreporting or misdiagnosis of subclinical or mild infections. Additionally, the use of high-resolution molecular tools, such as ICP4 sequencing and phylogenetic comparison, have proven to be essential not only for identifying circulating strains but also for uncovering hidden transmission chains. Implementing such molecular diagnostic approaches across Chile could inform targeted vaccination policies and improve the overall understanding of ILTV dynamics in both industrial and backyard poultry systems.

## 4. Conclusions

This study provides the first molecular evidence of ILTV circulation in unvaccinated backyard poultry flocks in southern Chile, with phylogenetic analysis revealing close genetic similarity to TCO vaccine strains. The detection of vaccine-derived sequences in unvaccinated birds suggests indirect transmission, likely due to inadequate biosecurity or environmental persistence. These findings highlight a gap between official surveillance data and field circulation, emphasizing the need for improved molecular monitoring in non-commercial settings. qPCR targeting the ICP4 gene, combined with phylogenetic analysis, proved effective for variant detection and characterization. Continued genomic surveillance and the integration of molecular tools into national health strategies are essential to better understand ILTV dynamics and inform vaccination policies.

## Figures and Tables

**Table 1:** Set of primers used in qPCR. ICP4-1 and ICP4-2 primers were designed in this study while UL15 primers were obtained elsewhere.

Primer	Sequence (5′-3′)	Amplicon
ICP4-1-Fw	ACTGATAGCTTTTCGTACAGCACG	604 bp
ICP4-1-Rv	CATCGGGACATTCTCCAGGTAGCA	
ICP4-2-Fw	CTTCAGACTCCAGCTCATCTG	662 bp
ICP4-2-Rv	AGTCATGCGTCTATGGCGTTGAC	
UL15a-Fw	TTGCTGTGCTATTTCGCGTG	113 bp
UL15a-Rv	GTAAATCGTTTAGTGCGGCAT	

## Data Availability

The original contributions presented in this study are included in the article, the newly generated data are available in the NCBI database.

## Supplementary Materials

The following supporting information can be downloaded at: https://www.mdpi.com/article/10.3390/ani15111623/s1, Table S1: List of sequences aligned against the consensus alignment.
