# Supplementary material for: Development of a qPCR Tool for Detection, Quantification, and Molecular Characterization of Infectious Laryngotracheitis Virus Variants in Chile from 2019 to 2023
Source: Animals (Basel). 2025 May 31;15(11):1623. doi: 10.3390/ani15111623 (PMC12153605; doi:10.3390/ani15111623)
Supplement: Supplementary file 1 [file animals-15-01623-s001.zip › animals-3609166-supplementary.pdf]

Supplementary table S1. List of sequences aligned against the consensus alignment.

NCBI access number	Name
JN542533	Strain 1874C5
JN542534	Strain USDA
JN542535	Strain 81658
JN542536	Strain 63140-C-08-BR
JN580312	TCO IVAX
JN580313	CEO TRVX
JN580314	TCO High passage
JN580315	TCO Low passage
JN580316	CEO High passage
JN580317	CEO Low passage
MN518177	USDA09-2019
MN784692	Vbc-112219
MN784693	USA vModKLO
KY423284	USA A489
KP677882	Strain Poulvac ILT
KP677881	Strain Nobilis Laringovac
HQ630064	Serva
JQ083493	LT Blen
JQ083494.2	Laryngo Vac
MN792995	Isolate vKLO
JX458822	Strain LJS09

JX458823	Strain WG
JX458824	Strain K317
MT876619	Strain ck-CH-LHLJ-120305
MK905886	Strain LJL180301
MK905887	Strain LSN1803A
JN596962	SA2
JN596963	A20
JN804826	Strain ACC78
JN804827	Strain CL9
JX646898	V1-99
JX646899	CSW-1
KR822401	ILTV class 10
MF156847	CSW-v1-99 isolate 1
MF156848	CSW-v1-99 isolate 8
MF156849	CSW-v1-99 isolate 12
MF156850	CSW-v1-99 isolate 15
MF156851	CSW-v1-99 isolate 23
MF156852	CSW-v1-99 isolate 27
MK894996	Strain 29-19
MK894997	Strain 109-19
MK894998	Strain 138-19
MK894999	Strain 157-19
MK895000	Strain 237-19

MK895001	Strain 238-19
MK895002	Strain 319-19
MK895003	Strain 358-19
MN335811	Strain 7b
KU128407	Strain o
MF405079	Strain CK-tatarstan 2009-1643
MF405080	Strain CK-penza-2013-2701
KP677883	Strain 193435-07
KP677884	Strain 757-11
KP677885	Strain 4787-80
MH937564	Strain 0206-14-Ko
MH937565	Strain 30678-14-Ko
MH937566	Strain 40798-10-Ko
MG775218	VFAR-043
MF417807	Strain S2.816
MF417808	Strain J2
KX165320	Strain BdORFC
KX165321	Strain GdORFC
MF417809	Strain 3.26.90
MF417810	Strain 6-48-88
MF417811	Strain 14.939
MT797242	Isolate CAN/AB-S45
MT797248	Isolate CAN/AB-T85

MT797246	Isolate CAN/AB-S77
MT797250	Isolate CAN/QC-1990662
MT797241	Isolate CAN/AB-S42
MT797243	Isolate CAN/AB-S50
MT797244	Isolate CAN/AB-S61
OL661344	Isolate CAN/QC-2307414
MT797239	Isolate CAN/AB-15A
OK573459	Isolate CAN/ON-246224
OK624781	Isolate CAN/ON-2462242
MT797249	Isolate CAN/BC-10-1122
ON598586	Isolate CAN/QC-2551439
OK646550	Isolate CAN/QC-2301711
OL354140	Isolate CAN/QC-2236832
MT797252	Isolate CAN/QC-2175807
ON598585	Isolate CAN/QC-2470159
MT797245	Isolate CAN/AB-S63
MT797251	Isolate CAN/QC-2154822
MT797240	Isolate CAN/AB-S20
NC006623	ILTV reference Seq. Bioproject: PRJNA485481
